# Microscale dynamics promote segregated denitrification in diatom aggregates sinking slowly in bulk oxygenated seawater

**DOI:** 10.1038/s43247-023-00935-x

**Published:** 2023-07-28

**Authors:** Davide Ciccarese, Omar Tantawi, Irene H. Zhang, Desiree Plata, Andrew R. Babbin

**Affiliations:** 1 Department of Earth, Atmospheric & Planetary Sciences, Massachusetts Institute of Technology, Cambridge, MA USA; 2 Department of Civil and Environmental Engineering, Massachusetts Institute of Technology, Cambridge, MA USA; 3 Program in Microbiology, Massachusetts Institute of Technology, Cambridge, MA USA

**Keywords:** Carbon cycle, Biooceanography, Ecology

## Abstract

Sinking marine particles drive the biological pump that naturally sequesters carbon from the atmosphere. Despite their small size, the compartmentalized nature of particles promotes intense localized metabolic activity by their bacterial colonizers. Yet the mechanisms promoting the onset of denitrification, a metabolism that arises once oxygen is limiting, remain to be established. Here we show experimentally that slow sinking aggregates composed of marine diatoms—important primary producers for global carbon export—support active denitrification even among bulk oxygenated water typically thought to exclude anaerobic metabolisms. Denitrification occurs at anoxic microsites distributed throughout a particle and within microns of a particle’s boundary, and fluorescence-reporting bacteria show nitrite can be released into the water column due to segregated dissimilatory reduction of nitrate and nitrite. Examining intact and broken diatoms as organic sources, we show slowly leaking cells promote more bacterial growth, allow particles to have lower oxygen, and generally support greater denitrification.

## Abstract (web summary)

Isolates of marine denitrifying bacteria actively reduce nitrate and nitrite at anoxic microsites on marine snow particles consisting of intact and broken diatom cells even under oxygenated water conditions, suggest results from a model experimental system.

## Introduction

The microbial metabolism of denitrification exerts a principal control on the ocean’s global productivity as fixed, bio-available nitrogen limits primary production across most of the global ocean. This anaerobic pathway conducted by specialized organisms is characterized by the stepwise reduction of nitrate through nitrite, nitric oxide, and nitrous oxide to dinitrogen gas. Denitrification occurs when dissolved oxygen concentrations are sufficiently low as to minimize the oxygen poisoning of the enzymes themselves and many heterotrophic denitrifying organisms facultatively switch from solely respiring aerobically^1^. Because of the dependence on limited oxygen availability, marine denitrification has classically been investigated in sediments and the ocean’s oxygen deficient zones (ODZs) of the eastern tropical Pacific and Arabian Sea^2^. Further, global biogeochemical models typically only consider these two marine settings when closing the fixed nitrogen budget^3^.

Sinking marine particles, however, have been largely excluded from consideration as potential denitrification hotspots of global significance despite a growing recognition of the importance of particles for marine biogeochemistry. This marine snow combines the essential features that give rise to denitrification in sediments and ODZs: physical confinement acts to exert a diffusion limit of oxygen supply from the oxygenated bulk and particles are locally enriched in organic matter. Physical confinement coupled to being the carbon source itself combine to make marine particles likely candidates for hosting denitrification^4–7^, but unlike ODZs and sediments, one distributed across the global water column. Bianchi et al. modeled how particles might develop given dynamic aggregation and disaggregation to show that the region where denitrification is permitted expands well beyond the ODZs^7^. Yet, real marine particles, to date, are difficult to sample intact and in sufficient quantities to measure metabolites, chemistry, or microbial activity because of their ephemeral and dispersive nature throughout the ocean.

Still, much progress has been made in recent years on denitrification in marine snow. Ploug and Klawonn et al. showed anoxia only develops in the core of a particle when the bulk oxygen is below 10% saturation^8,9^. Klawonn et al. demonstrated empirically and Bianchi et al. numerically that aggregates and particles exhibit a one-dimensional dependence of intraparticle oxygen concentrations along the radius from the center of the particle^9,10^. With these analyses and models, they showed anaerobic cores can arise in large particles even when oxygen concentrations in the bulk are high, governed by diffusion limitation from the boundary^11^. However, the distribution of the bacterial biomass and activity in real particles is heterogeneous, and local physiochemical conditions can change at much finer scales as colonies develop^12^. As a result, there is potential for anaerobic microsites to emerge beyond just particle centers. Instead, as colonies grow, they consume oxygen and can even decrease standing concentrations near to the particle boundary^13^, the consequence being that the interplay between bulk and particle chemistry happens at much shorter distance from the boundary layer than implied by one-dimensional idealized models. These fine scales in turn can facilitate the release of particle-based metabolic products to bulk seawater as the diffusional distance to the bulk is orders of magnitude shorter.

Numerous studies have reported particle-based denitrification on the basis of gene abundance and activity, directly measured rates, empirical biogeochemical profiles, and numerical modeling. Stief et al. examined compact diatom aggregates to show measurable denitrification rates at bulk oxygen levels 1–2 orders of magnitude greater than inhibitory concentrations^5^. They further showed the diatoms themselves act as a significant nitrate source for denitrification from intracellular reserves of fixed nitrogen, decoupling ambient nitrate supply from denitrification activity. Yet, they could not address the question of whether less compact aggregates develop anoxia and promote denitrification. Stief et al. showed sinking zooplankton carcasses can increase denitrification rates sizably, up to 40% in hypoxic yet not anoxic water^14^. Using entirely different methods, Fuchsman et al. found a shallow peak of biogenic N_2_ gas in hypoxic waters in the Black Sea following a transient high organic matter event during blooms^15^. This, coupled to the isotopic signature of the N_2_ gas lent itself to microsite denitrification in particles, with equal contribution of nitrogen sourced from the ambient environment and from intraparticle remineralization as the denitrified material. Together, these studies suggest that particle dentification can be widespread with the organic matter supporting both the carbon and nitrogen requirements of denitrifiers^14,15^. Yet, the governing biophysical mechanisms are not yet fully understood. For instance, and importantly, whether denitrification emerges only in the central core of a particle as suggested by Klawonn et al. and Bianchi et al. or whether it manifests closer to the edge due to microcolony development per Smriga et al. yet in a system with organic carbon supplied at low rates remains a question^5,10,13^. By revealing the highly resolved landscape of denitrification in particles, we can unveil how small scale anoxic sites form and how denitrification can occur in marine particles distributed globally in the well-oxygenated ocean. Moreover, our characterization of the microenvironment dynamics with unprecedented spatiotemporal resolution enhances our understanding of these processes.

In the ocean, there are two dominant pathways of marine snow generation: the consumption and repackaging of phytoplankton into fecal pellets by zooplankton^16,17^ and the spontaneous clumping of aggregates, which is particularly common during blooms^17,18^. Each represents an endmember of the spectrum of carbon availability dictated by the intactness of the diatom cells, with the former releasing all the dissolved carbon rapidly in a pulse and the latter maintaining more carbon for longer via a slow leak. We hypothesize that the broken diatoms should induce a *boom-and-bust* scenario for facultative copiotrophs able to respond rapidly to the nutrient pulse whereas the intact cells should permit a slower but ultimately more abundant community to develop. Moreover, the oxygen drawn down inside the particle should be more intense from intact cells because the organic nutrients are better retained. Combined, we anticipate that the intact diatoms would lead to less dissolved organic carbon (DOC) leaked to the water column than the broken diatoms. Yet, how readily denitrification can rise within this system will depend on the speed of microbial growth on natural phytoplankton exudates compared to the flushing rate by diffusion at the scale of the bacterial microcolonies themselves. Due to the ephemeral nature of sinking particles and their optical inaccessibility that prevents three-dimensional visualization, the dynamics of how microscale processes impact the macroscale remain largely unexplored empirically.

Here we developed a laboratory system that replicates marine snow in the ocean, composed of marine organic matter that moves through the water column. We amended the system and fluidic device of Smriga et al. for marine relevance. Instead of supplying rich nutrients externally, bulk inorganic nutrients concentrations are comparable to seawater levels and organic matter is supplied exclusively by phytoplankton within the particle. We show how readily oxygen concentrations are reduced locally in a particle, determine where denitrification enzymes are turned on and activity distributed within a particle, and elucidate how microscale dynamics influence the bulk water column chemistry. We explored both conditions of marine particle generation, by seeding our experiments with broken diatoms and intact diatoms to approximate repackaging by zooplankton and phytoplankton aggregate formation, respectively. The novel method employed here overcomes the limitation of inaccessible, ephemeral, and non-transparent particles to open a window into the highly spatiotemporal dependent scale of heterogeneous biomass development, microscale oxygen consumption, and bulk chemistry evolution.

## Results and discussion

### Selective pressures within the phycosphere

To generate an inoculum with which to seed our particle experiments, we first had to isolate organisms from the milieu of the phycosphere^19^. We successfully isolated a number of bacterial members of the community, many of which tested positive for the ability to reduce nitrate and nitrite to N_2_ gas. In all, we seeded our particles with a mixed assemblage of five bacterial species with denitrification ability, each representative of different genera: *Alteromonas*, *Marinobacter*, *Phaeobacter*, *Idiomarina*, and *Virgibacillus*. The prevalence of facultative denitrifiers within the phycosphere emphasizes the potential of localized denitrification in phytoplankton-derived marine snow. As a carbon source, we used the chain-forming centric diatom *Chaetoceros affinis*, a globally important phytoplankton found in productive waters^20–23^, and one that produces extracellular polysaccharides and releases carbohydrates and amino acids throughout its lifecycle^23^. This organism has also been shown to attract bacteria to cultivate its own phycosphere^24^.

**Fig. 1:**
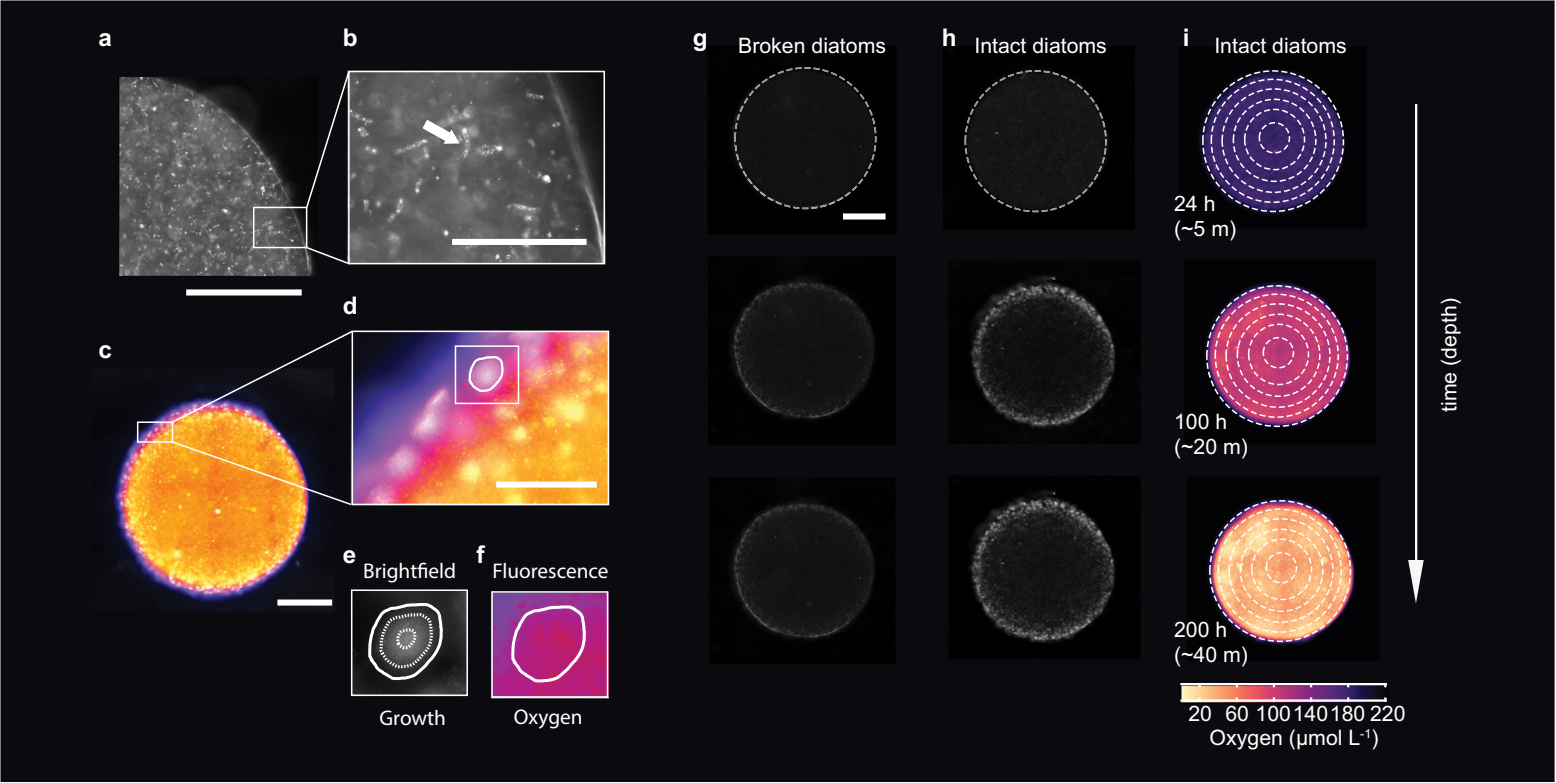
Particles seeded with diatoms and their oxygen evolution during sinking. **a** Representative microscopy image of a particle displaying the intact diatoms seeded within the hydrogel and the resulting bacterial colony development. The white scale bar denotes 1000 µm. **b** Close up of intact diatoms within the hydrogel particle. A white arrow indicates an intact diatom chain. The white scale bar denotes 250 µm. **c** False colored particle displaying the change in oxygen concentration across the particle as measured by fluorescent reporting nanoparticles. **d** Close up of the edge of a particle showing a steep gradient of oxygen at the edge. One colony is highlighted with a white contour. The white scale bar denotes 250 µm. **e** The area of each colony is measured at every time point, using brightfield images. The dashed lines denote the sizes at 24, 100, and 200 h. **f** The oxygen consumed within each colony is measured by quantified the fluorescence of a nanosensor encased within the microcolony’s biomass. **g**, **h** Brightfield images of particles seeded with broken diatoms and intact diatoms, respectively. Visualization of the bacterial biomass accumulation is shown for three time points, equivalent to 5 m, 20 m and 40 m. **i** Increase of nanosensor fluorescence as the oxygen is consumed during sinking. The white scale bar in (**g**) corresponds to all particles shown in (**g**–**i**) and denotes 1000 µm.

To approximate in-situ marine snow particles, we suspended the marine diatom culture in agarose (Fig. 1a, b). The agarose here is minimally bio-available yet acts to stabilize the particle and keep it together, similar to various transparent exopolymer (polysaccharide) particles (TEP) in the ocean^25–29^. *C. affinis* produces much TEP, particularly for its size^30^. The culture was mixed with an assemblage of organisms isolated from the phycosphere itself, each shown to have facultatively anaerobic growth potential. Suspending the mixed cultures in agarose in such a fashion replicates the way diatomaceous marine snow would form, i.e., the clumping of cells with TEP^25,31–34^ and their representative phycospheres^24,35–38^ being distributed throughout. We lastly suspended oxygen-sensitive nanoparticles in the aggregates to track precisely the microscale dynamic consumption of oxygen throughout the aggregates (Fig. 1b) and the local consumption within each expanding individual bacterial colony (Fig. 1c). The agarose disks were sandwiched between two impermeable glass plates and seawater was flown around them at a speed of 4.8 m d^−1^, similar to sinking speeds in the ocean of loose diatom flocculated particles^31,39–42^. The particles generated, 1.5 mm in radius, were imaged every hour for over 10 days with epifluorescence microscope and the seawater flowing around the particles was sampled once daily for bulk chemistry (nitrate, nitrite, and total dissolved organic carbon).

Particle abundances, sizes, and sinking speeds remain an important and contested topic in marine biogeochemistry^43^, with real marine snow exhibiting complex structure and diverse composition^44,45^. Most particles sink slowly^46^, on order of 1–10 m d^−1^ although some large and dense particles can sink very quickly, up to hundreds of meters per day^47^. Yet, the data of real ocean sinking speeds as a function of particle size do not form a coherent relationship^48^. Rather, the large variability in direct measurements suggests the oceans consist of particles ranging between 0.1 and a few mm in size, any of which can sink between 1 and 1000 m per day^49^. The particles investigated here, at 1.5 mm in radius sinking at 5 m d^−1^ fall within this environmental range. These large particles are also quite important in the ocean. From the global compilation by Clements et al., large particles >1 mm in size comprise only up to about 0.3% of total particles at the export horizon (mean of 0.16%) but importantly up to 65% of the total particle biovolume (mean of 48%)^50^. Given our focus on intraparticle dynamics and the relationship with integrity of the diatom cells, particle size and sinking speeds were not varied among the treatments. Yet, these two physical variables of size and velocity are critically important in the ocean relating to the biological pump’s transfer efficiency^51,52^. Notably, larger, faster particles contribute greatly to the mass flux in the ocean whereas smaller, slower ones dominate in number^53^.

**Fig. 2:**
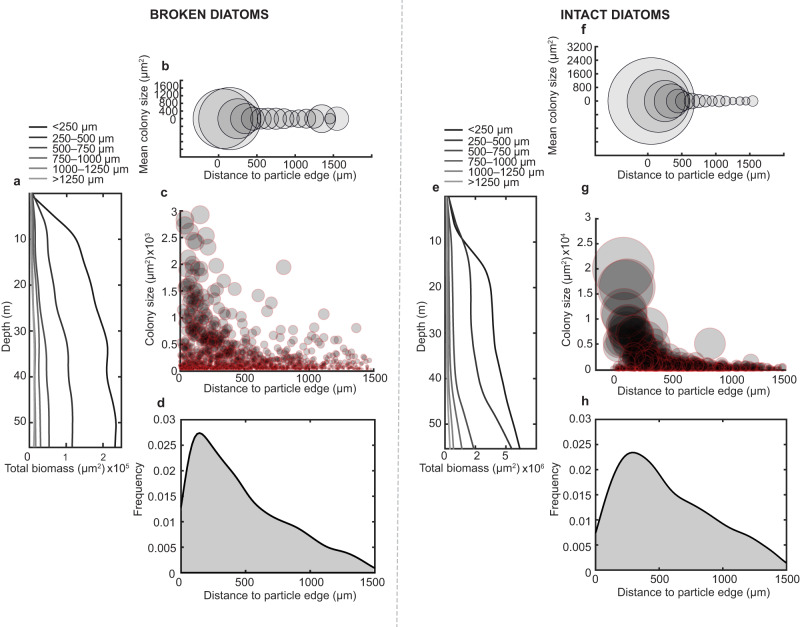
Broken and intact diatoms drive spatially heterogeneous biomass accumulation. **a** Translated depth profile of the cumulative biomass productivity at different distances from the edge of particles seeded with broken diatoms (*n* = 4). **b** Mean colony size of particles seeded with broken diatoms (*n* = 4) at different locations within the particles. **c** Decrease of colony size away from the edge of particles seeded with broken diatoms (*n* = 4), each dot size is proportional to the size of the colony. **d** Probability density function of the distribution of microcolonies along the radius of particles seeded with broken diatoms (*n* = 4). **e**–**h** The same as (**a**–**d**) but using intact diatoms as the carbon source instead of broken ones.

When diatom-bacterial aggregates form in the water column, interactions at the microscale unfold rapidly^16,18,19,29^. The results of these interactions impact the efflux of phytoplankton exudates and metabolic products that ultimately influence water column biogeochemistry^27,28^. The diatoms leak their organic contents that serve as nutrients for bacterial heterotrophs across an aggregate^17,35,54–56^. These diverse bacteria in turn can interact to form self-engineered compartmentalized spaces that optimize the supply of necessary nutrients via diffusion^6,8,9,11,16,17,35,40,57,58^. Yet, if bacteria cannot grow fast enough to intercept the labile carbon leaking from the phytoplankton around them, the dissolved organic carbon resource is lost from the aggregate environment^59,60^. To understand how local gradients drive bacterial productivity across space and its link to the larger scale processes, we quantified the biomass accumulation with respect to the distance to edge of a particle (Fig. 2). Increased growth manifested closer to the edge than the center in both the intact and broken diatom conditions (Fig. 2a, e) because even though the source of organic carbon is distributed throughout the particle, the oxidants (oxygen and nitrate) are resupplied from the bulk. As such, it is the oxidants that limited the colony development. Yet, the integrity of the diatoms further played a role as the broken diatoms do not permit as much growth as their intact counterparts (Fig. 2b, f). This is likely because the initial organic reserves contained with the cells were released too quickly, resulting in their ultimate loss to the bulk seawater rather than their bacterial consumption and recycling.

The distributions of biomass at the end of the experiments in both broken and intact diatom particles showed a comparable decay with size moving inward of the particles (Fig. 2c, g), with the colonies closest to the oxygenated edge being largest. Yet, the intact diatoms supported much more growth (approximately double) of the colonies closest to the edge than the broken diatoms did. The number of colonies that developed in particles with broken diatoms was slightly higher in number closer to the edge, compared to those that emerged in the particles seeded with intact phytoplankton (Fig. 2d, h). The larger colonies supported by the intact diatoms did not permit as many colonies to develop because of competition for space and resources.

**Fig. 3:**
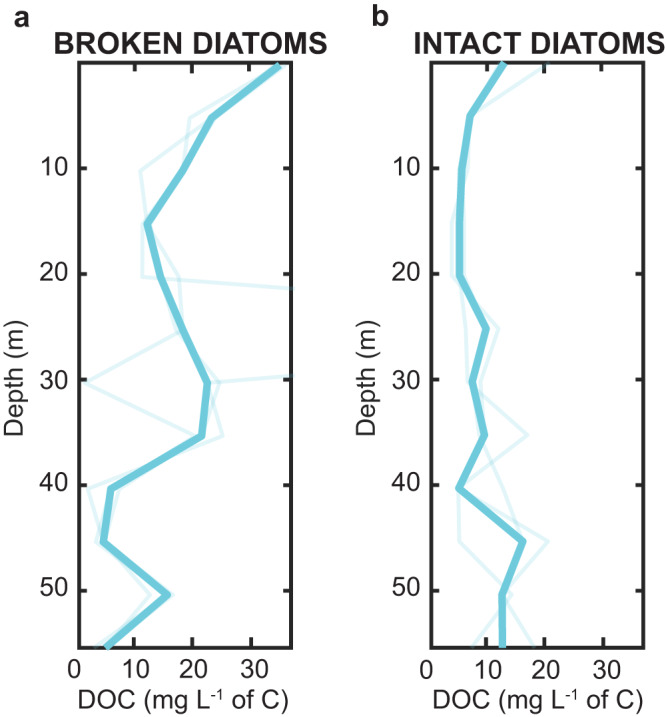
Dissolved organic carbon profiles of diatom-seeded particles. **a** Depth profiles of dissolved organic carbon from particles seeded with broken diatoms. The thicker line is the mean of three replicate devices each containing *n* = 4 biological replicates, 12 particles in total. **b** same as (**a**), but of particles seeded with intact diatoms.

By monitoring the dissolved organic carbon that escaped from the particle into the bulk seawater, we can relate biomass growth dynamics with the bulk carbon retained within a particle that is not lost to the flowing bulk environment. The particles seeded with broken diatoms (Fig. 3a) initially leaked a higher (two-sample t-test, *p* = 0.003) amount of carbon (28.0 ± 0.7 mg L^−1^ of C mean ± std) compared to the particles seeded with intact diatoms (14 ± 3.6 mg L^−1^ of C mean ± std). Three-fold more DOC escaped the particle initially, i.e., the analog of the export horizon, thus indicating less organic carbon availability maintained locally within the particle to support later bacterial biomass accumulation. Yet, notably, particles seeded with intact diatoms showed less release of DOC to the bulk (Fig. 3b), maintaining similar effluxes across the entire lifetime of the particle, retaining more carbon, and leading to greater ultimate bacterial biomass. The bacterial cells could intercept the carbon faster than the diatoms leak it out. The impact of these two different modes of organic leakage can be profound: the leakier broken cells will lead to less export of organic matter via the gravitational pump and greater remineralization in shallower waters. This in turn will limit the sequestration of carbon into the deep ocean. Conversely, intact phytoplankton not only retain their carbon better, but the bacteria that comprise their phycosphere are able to recycle a larger fraction of the carbon that leaks and maintain it in the sinking particle, permitting higher export fluxes to deeper depth and higher magnitudes of sequestration for longer times.

### A highly localized oxygen landscape driven by divergent carbon reserves

Oxygen is perhaps one of the most dynamic state variables across a marine snow particle actively being colonized by heterotrophs. As such bacteria consume the oxygen around them, they begin to self-limit their own aerobic growth. At the scales of the microorganisms themselves, this can happen across a particle or across even a microcolony itself (Supplementary Figs. 5–8) For facultative anaerobes, this highly localized oxygen landscape translates to a locally heterogeneous metabolic population and increases in niche functions^13,61^. Indeed, the evolution of a particle’s oxygen landscape is apparent in these experiments (Supplementary Figs. 5, 7). At the early time points (analog to depth in the real ocean) of the experiments, the vast majority of colonies were well-oxygenated at concentrations close to the saturation conditions of the bulk seawater flowing around the particle. As the particle descended, however, these colonies became progressively less oxygenated (~20 m; Supplementary Figs. 5, 7). However, this decrease in oxygen concentration caused the organisms themselves to respond dynamically and slow down via their gene regulation, which in turn permitted the oxygen concentrations to slightly increase by ~50 µmol L^−1^ before continuing to decrease. This stage coincided with very little change of oxygen across a particle or microbial growth (Supplementary Figs. 5–8). At the end of the progression, the colonies across the particle were close to fully anoxic, with the entire microbial metabolism supported solely by the leaking carbon from diatom cells. Interestingly, both the colonies in close proximity to the particle edge and those nearer to the center could become anoxic in this system, even if those further interior could more readily experience anoxia due to the greater distance to resupply oxygen diffusively from the bulk seawater. Notably, the oxygen concentrations observed by the colonies themselves were nearly indistinguishable from the concentrations at the equivalent particle radius where no colonies develop, even though the colonies themselves are the sinks of oxygen (Supplementary Figs. 5–8). This occurred because at the very small spatial scales, the colony’s oxygen equilibrates quickly (less than a second) with its immediate surroundings via diffusion.

**Fig. 4:**
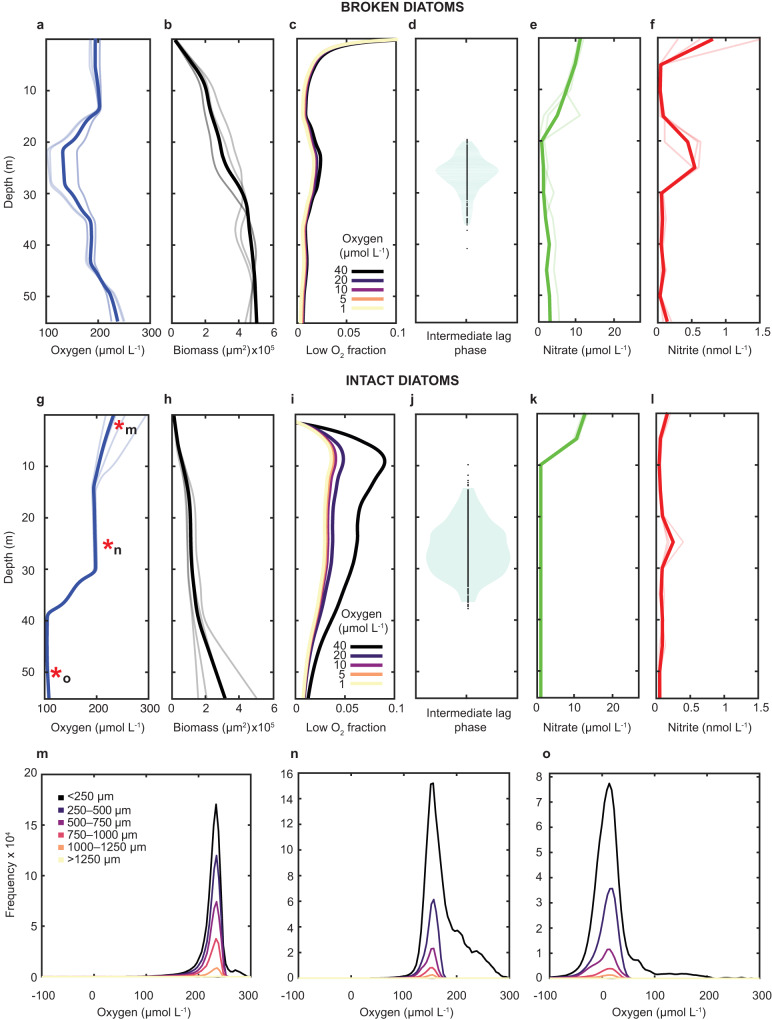
Oxygen, biomass accumulation, nitrate and nitrite dynamics. For particles seeded with broken diatoms: **a** Oxygen change with translated depth at the colony level due to colony respiration. The thick line is the mean value of four particle replicates, each indicated with a lighter color. **b** Biomass accumulation as determined by colony cross-sectional area. **c** Fraction of total colony area that experiences different oxygen conditions. Each line shows the area where oxygen is less than the shown value. **d** Intermediate lag phase distribution. **e** Nitrate evolution in the bulk seawater. **f** Nitrite evolution in the bulk seawater. For particles seeded with intact diatoms, **g**–**l** Correspond to the broken diatom counterparts. **m**–**o** Histograms of oxygen concentrations within colonies at 5, 25, and 50 m, respectively. The colonies are distinguished by their locations in the particles as distance from particle edge. Asterisks in (**g**) denote the time points of these panels.

In particles seeded with broken diatoms, approximately one mode of marine snow generation by zooplankton repackaging, the microcolony respiration caused oxygen to reach its lowest concentration at 20–30 m effective depth (Fig. 4a) before oxygen increased again as the biomass entered stationary phase (Fig. 4b). This stage coincided with a short plateau of relatively constant average oxygen of 145 ± 50 µmol L^−1^ (Fig. 4a) across the colonies. This intermediate plateau in oxygen consumption corresponded to a temporary increase of local biomass experiencing (Fig. 4c) hypoxic (<40 µmol L^−1^), microoxic (<10 µmol L^−1^), suboxic (<5 µmol L^−1^) and nanoxic (<1 µmol L^−1^) conditions^62^. That is, even though the median colony did not change in its observed oxygen concentration, the number of colonies (although still a vast minority of cells) experiencing localized oxygen low enough to permit denitrification did increase.

For facultative heterotrophs, a decrease in oxygen levels activates a regulatory network that controls denitrification genes, leading to changes in growth patterns^63–66^. This transition to anoxic conditions has been observed to trigger a characteristic diauxic phase in denitrifying organisms^67–69^. In addition, experimental evidence shows that only a fraction of cells can express the *nirS* gene before all the oxygen is depleted^20,21^. Models have also suggested that there is a low probability of initiating the transcription of the *nirS* gene^70^ ahead of the onset of anoxia. Moreover, the delay in synthesizing the nitrite reductase has also been identified as a form of bet-hedging to avoid further energy investment in cells undergoing sudden anoxia^71^. These previous studies support our experimental observation of an intermediate stage of slow colony growth occurring over 9.6 ± 4.1 m of sinking (mean ± std), which indicated the time required for the regulatory system to synthesize the necessary denitrification enzymes as a response to the change in local oxygen availability. This intermediate plateau of the growth notably had an impact in the water column, giving rise to a simultaneous visible nitrite peak at the same depth. When finally, the bacteria reached late stationary phase, having exhausted all labile carbon resources at >30 m depth (Fig. 4c), oxygen penetrated again within the particles and each colony equilibrated with oxygen in bulk seawater. Notably, there were many hypoxic, suboxic, and anoxic colonies initially in this experiment with broken diatoms, corresponding to the greatest rate of carbon leakage from the phytoplankton (and the highest nitrite concentrations measured).

Particles seeded with intact diatom aggregates, however, display a similar yet importantly different metabolic evolution. After an initial decrease of oxygen driven by the initial exponential growth of the biomass accumulation (Fig. 4g, h), colonies subsisting under local hypoxic conditions expanded about two-fold more than the biomass that experienced less oxygen (Fig. 4i). After an initial exponential growth phase, the bacteria slowed and entered an intermediate lag phase contemporaneous with the intermediate intraparticle oxygen plateau. The intermediate lag phase of biomass growth, observed in particles seeded with broken diatoms, occurred when the colonies reached a local oxygen concentration of around 145 µmol L^−1^, indicating a decrease in oxygen availability but not anoxia. These cells also displayed a wider lag phase (translated to depth) distribution (12.7 ± 5.0 m mean ± std) compared to those in association with broken diatoms. At the end of this intermediate lag phase, the colonies restarted their metabolism and entered a second exponential growth phase, now nearly fully anoxic, and still supported by the available carbon continuously provided by the extant diatom cells. Like the particles seeded with broken diatoms, these too indicated that the metabolic activities were such that nitrite only accumulated in the bulk water at the peak of the lag phase distribution. The particles seeded with intact diatoms showed a significantly longer lag phase compared to the particles seeded with broken diatoms (two-sample t-test, *p* = 1.3 × 10^−22^).

When comparing the experiments conducted with intact diatoms and their broken counterparts, general trends emerged with some notable distinctions. Under both scenarios, multiple microbial growth phases appeared that cause oxygen to be reduced at the scale of a colony. The same opposing forces at play in an ODZ—biological consumption of oxygen and physical resupply—yielded a time (ergo, depth) at which oxygen was a minimum. Oxygen was consumed more rapidly initially with the broken cells leaking much of their organic contents as a pulse, stimulating growth but leaking DOC into the surrounding seawater. An initial leak of nitrite was also observed here in all broken diatom experiments (Fig. 4f). However, colony growth stagnated quickly as the organic matter contained within the phytoplankton was not retained in the particle. In contrast, intact cells permitted a slow leakage of organic material that delayed colonization but ultimately created an environment sustaining higher microbial biomass and thus higher oxygen drawdown and rates of denitrification. In fact, both broken and intact diatoms yielded similar progressions for the first half of the experiments up to the point of the intermediate lag phase that coincided with oxygen depletion to ~145 µmol L^−1^, but in the broken trials, labile organic matter was exhausted earlier whereas in the intact ones, the bacteria at the edge had sufficient resources of both reductant and oxidant to continue to colonize and thrive. The only cells growing at the latest time points in the intact experiments were those at the edge, but they were growing aerobically given the contraction of the suboxic area and constant size of the anoxic zone.

### Spatial segregation of nitrate and nitrite reduction

To understand more mechanistically how denitrification in marine snow manifests, the impacts for water column chemistry, and link gene expression with location across the particle and highly localized oxygen conditions, we additionally mixed diatom cultures with denitrification-reporting strains of the ubiquitous bacterium and facultative denitrifier *Pseudomonas aeruginosa*, an organism with the full complement of enzymes to reduce nitrate to dinitrogen. This organism is found in the ocean itself^72–74^ but is typically studied as an opportunistic human pathogen, and here it was used as a representative model for similar marine denitrifiers, many of which are also gamma proteobacteria (e.g., *Marinobacter*, *Alteromonas*, and *Idiomarina* spp.)^75,76^.

**Fig. 5:**
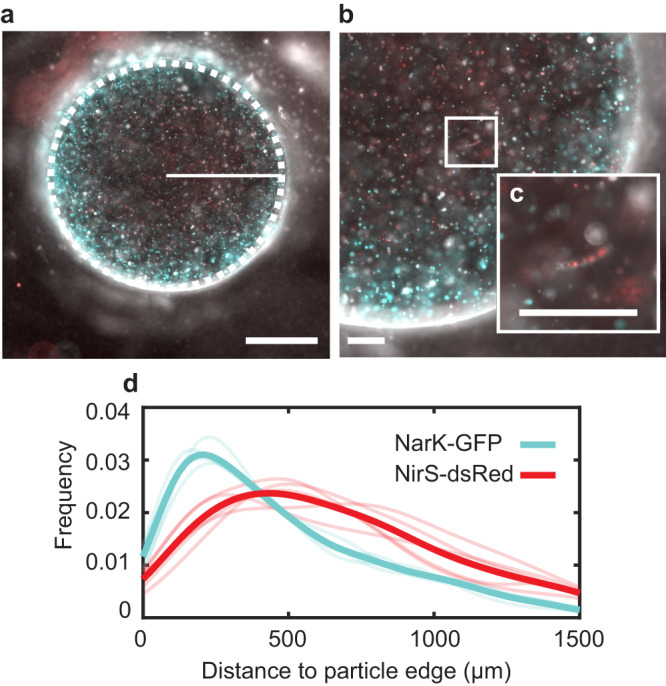
Spatial distribution of NarK and NirS gene expression and diatoms. **a** Particle seeded with *Pseudomonas aeruginosa* PAO1 NarK-GFP, *P. aeruginosa* PAO1 NirS-dsRed and *Chaetoceros affinis*, the dashed white line identifies the particle edge while the solid line indicates the radial sampling of the gene expression within the particle. The white scale bar denotes 1000 µm **b** Close up of one-quarter of the particle showing the diatoms and denitrifying colonies. The white scale bar denotes 250 µm. **c** Inset shows in detail the intact diatom within the particle with the chlorophyll fluorescing naturally. The white scale bar denotes 250 µm. **d** Probability density functions of the expression of NarK-GFP and NirS-dsRed shown for each replicate, with an average overlain with a thicker curve. For all panels, GFP fluorescence is indicated in cyan and dsRed in red. Natural chlorophyll fluorescence is also shown in red.

The different steps of denitrification are each subject to different regulation by oxygen and other stimuli. Typically, nitrate reduction, the first step of canonical denitrification, can manifest at a higher oxygen tolerance than later steps, whereas the ultimate step of nitrous oxide reduction, is the most severely inhibited by oxygen^1,77^. Under this framework, the spatial segregation of the denitrification steps across a particle can be anticipated because higher oxygen concentrations will be maintained at the periphery. Within our particle experiments harnessing *P. aeruginosa* as a laboratory model of real world marine denitrifiers, the diatoms supported growth of the bacterial colonies and the commensurate oxygen drawdown sufficient to activate denitrification as measured by fluorescent reporter constructs for the promoters of the nitrate or nitrite reductase enzymes (Fig. 5). The greatest intensity of nitrate reduction was found at 204 ± 34 µm (mean ± std) from the particle edge (median 360 µm) whereas that of nitrite reduction was more interior, 489 ± 146 µm from the edge (median 634 µm). Notably, nitrite reduction still did occur toward the edge of the particle, just reduced compared to nitrate reduction. This distribution of gene expression can directly impact how much nitrite leaks out of a particle, if the nitrite reducing organisms cannot keep up with the nitrate reducing (nitrite producing) ones. The same phenomenon of segregated denitrification manifested whether growth is supported by the large chain-forming centric diatom *C. affinis* or by the smaller pennate diatom *P. tricornutum* (Supplementary Fig. 9), albeit the gene expression maxima were slightly closer in space.

In both experiments with different diatom sources, active denitrification (both nitrate and nitrite reduction) was observed across the particle, and moreover, in both, nitrate reductase regulation was skewed toward the particle edge whereas nitrite reductase reached its peak regulation inward of the edge by a few 100 µm. We explain this as arising from the dual control of both higher oxygen and nitrate toward the periphery, activating nitrate reductase instead of nitrite reductase. Together with recent studies that have observed nitrification on particles^78^ or free-living nitrite oxidation^79–81^, our findings highlight that intraparticle oxygen gradients can support spatial differentiation of denitrification activity between nitrate and nitrite reduction. The result is a spatial niche differentiation, that can yield a significant impact on the bulk, as the nitrite product of the first step diffuses equally inward and outward. Whereas later denitrification steps can reduce the nitrite in the particle center, whatever diffuses outward can leak into the bulk environment and modify the water column chemistry and subsequent planktonic biological activity in response.

### Potential causes of nitrite accumulation in the oxygenated subsurface ocean

In all experimental conditions—intact or damaged diatoms paired with marine bacterial isolates, the natural xenic phycosphere, or the laboratory model *Pseudomonas*—a transient nitrite accumulation was observed. Because here time was an analog for depth, we can transpose the axis, the result of which is a layer of nitrite buildup that corresponded to ubiquitous primary nitrite maximum (PNM) across the world’s oceans. The measured increase in nitrite here was small (approx. a nanomole per liter), but this arose from just four biologically viable particles in the nearly 15 mL that flowed past them per daily measurement. The PNM has classically been considered to arise from light limited phytoplankton that are incapable of fully reducing nitrate to ammonium for assimilation^82^ and/or inefficient coupling of ammonium and nitrite oxidizers during light-inhibited nitrification^83^. In addition to previous non-mutually exclusive hypotheses, we focus here on the potential role of particle-based denitrification in contributing to the PNM.

**Fig. 6:**
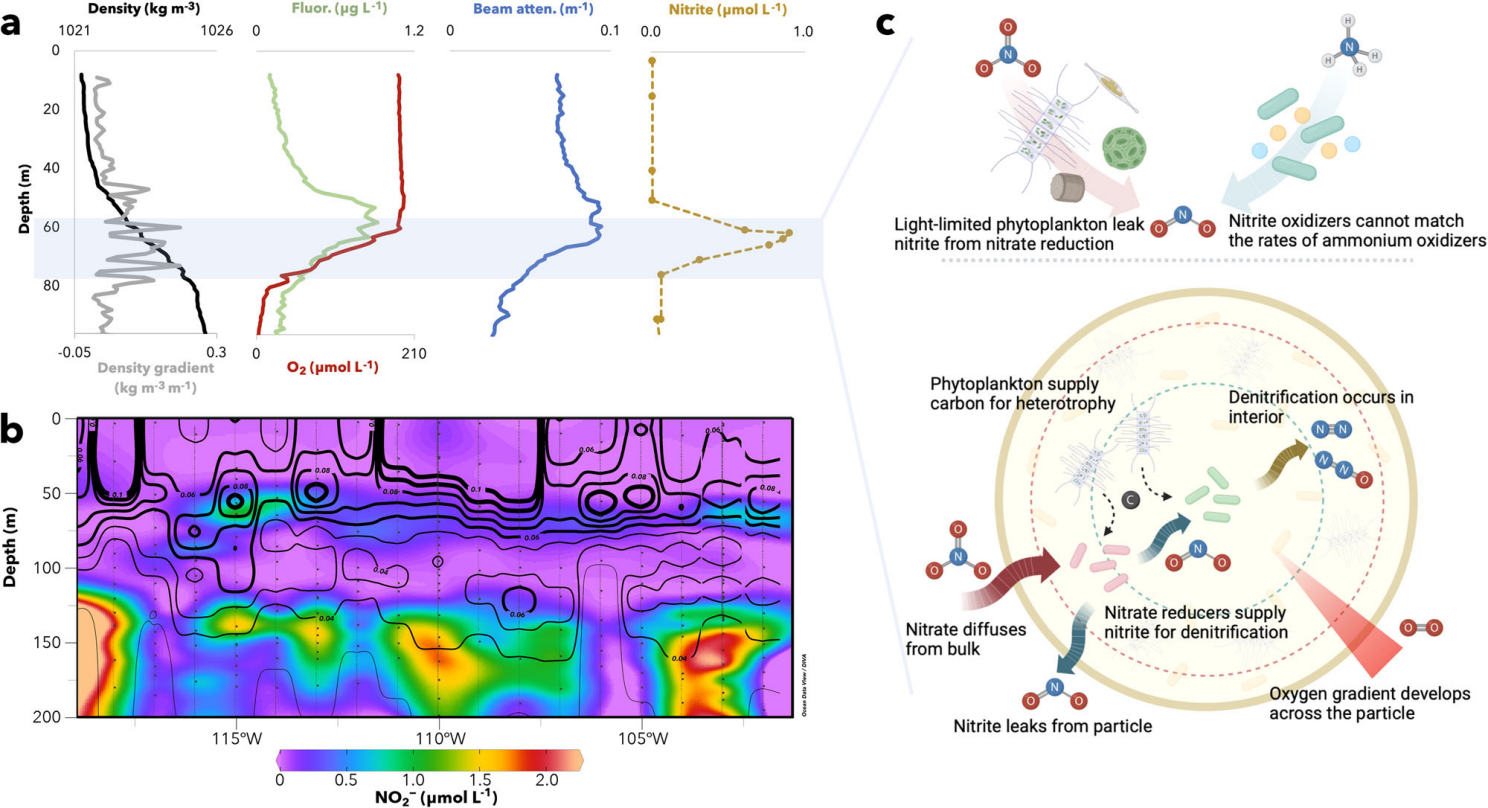
Particles and the primary nitrite maximum. **a** Profiles of water density (black), vertical density gradient (gray), fluorescence (green), oxygen (red), beam attenuation (blue), and nitrite (brown). The profiles show that the depths of accumulation at the primary nitrite maximum (noted in the blue box) align with the depths of maximal density gradient. Additionally, the primary nitrite maximum is just deeper than the fluorescence maximum of chlorophyll and aligns with maximal beam attenuation. **b** Zonal section of nitrite and beam attenuation from the R/V *Falkor* FK180624 cruise showing nitrite (color, in units of µmol L^−1^) and overlain by beam attenuation (black contours, in units of m^−1^). Data replotted from Cinay et al.^108^. **c** Potential pathways that lead to the production of nitrite at the primary nitrite maximum. The top two are well-established, light limited phytoplankton reducing nitrate inefficiently and ammonium oxidizers operating faster than nitrite oxidizers in nitrification. The bottom schematic describes the hypothetical denitrification within particles composed of phytoplankton detritus can be separated in space due to gradients in oxygen across a particle. Nitrate reduction occurs at the edge, permitting nitrite to diffuse inward and outward where it may be released to the water column.

The secondary nitrite maximum manifests from denitrification in the anoxic oxygen deficient zones of the eastern tropical Pacific and Arabian Sea wherein nitrate reduction outpaces nitrite consumption^84,85^. Here we suggest an analogous phenomenon in shallower subsurface waters in contributing to the ubiquitous PNM. A transect across the eastern tropical North Pacific revealed the PNM aligned with the highest particle concentrations (as inferred by increased beam attenuation) (Fig. 6). Moreover, stations where the particle concentrations were higher manifested more nitrite accumulation in this PNM layer (Fig. 6b). In terms of quantity, size, and total biovolume, the steep density gradient (pycnocline) retards sinking rates and traps particulate material^86–88^. These particles, derived from the surface, act to support increased heterotrophy in the shallow subsurface. Indeed, the depths of the steepest pycnocline aligned identically with the PNM (Fig. 6). Yet, the same conditions that trap particles at these depths would increase the rates of nitrification as well. Further, because the deep chlorophyll maximum is just above this layer, the contribution of light-limited phytoplankton harnessing insufficient photons to efficiently reduce nitrate to ammonium via nitrite cannot be excluded^89,90^. Thus, denitrification in particles need not cause the PNM, yet none of these nitrite production pathways at the PNM is mutually exclusive. Rather, a combined effect from all is the most likely reason due to the intrinsic alignment of the pycnocline with the deep chlorophyll maximum, i.e., the DCM naturally appears just below the mixed layer where cells have less light but greater access to nitrate. Given all these overlapping phenomena, we hypothesize that particle-based denitrification complements leaky phytoplankton and decoupled nitrification as a pathway leading to the rise of the primary nitrite maximum (Fig. 6c). However, to properly test this hypothesis and quantify what contribution particle denitrification can make on water column nitrite accumulation, much more work needs to be done both in situ and experimentally to elucidate the range of particle characteristics that can support denitrification.

### Broader implications

Taken together, the experiments combining different diatoms and bacteria highlighted how heterotrophic colonies grow and denitrification readily emerges within a marine snow particle supported solely by the organic lysate leaking from phytoplankton. Impressively, even the broken diatoms were not immediately and completely solubilized; rather, these particles, which are similar to what may be repackaged by zooplankton retained organic carbon far longer than the ~20-min time scale to flush the particle via diffusion. Intact diatoms retain their carbon even longer, permitting more growth, greater draw down of oxygen (in our experiments, two-fold more draw down, fully to anoxia), and correspondingly higher denitrification potential across over 10 days of sinking. The elucidation of this phenomenon coupled to the prevalence of marine particles globally and throughout the water column, can have far ranging implications for bulk ocean chemistry, the fixed nitrogen budget, and potentially nitrous oxide production. How these large budgets are resolved to include particle denitrification, and potentially other metabolisms favored in low-oxygen environments like diazotrophy, sulfate reduction, and methanogenesis, remains to be determined with substantial directed efforts^7^.

The marine fixed nitrogen budget has been debated for decades, whether it is balanced or not^91,92^, and what the climactic implications are for a global world if not. Additional pathways and mechanisms (like particles) have been revealed^10^, but the general budgets typically include fixed nitrogen loss by denitrification (and anammox) exclusively within sediments and in the ocean’s oxygen deficient zones. Here we have shown the microscale gradients developing in particles found throughout the water column enable them to readily support denitrification and other anaerobic metabolisms even when bulk oxygen is fully saturated with respect to the atmosphere. The expansion of denitrification capabilities on particles can dramatically increase the gross fixed nitrogen loss rates in the global oceans^7^, but just how much depends on the bulk ocean oxygen and microscale dynamics that permit denitrification to emerge. The observed development of denitrification throughout a particle even in 100% bulk saturated oxygen contrasts with modeled work of idealized particles. In Bianchi et al., individual colonies were not explicitly resolved. The consequence of our observations of individual colonies reported here is that denitrification can occur much closer to the particle edge and in a much wider range of bulk oxygen conditions. Just how much additional ocean volume where particle denitrification can occur, however, depends on future modeling work and data synthesis across the global oceans.

Whether the net loss rates, further suggesting a pronounced imbalance of sources and sinks, are in actuality greater than current estimates or are counterbalanced by additional diazotrophy in the oceans’ subsurface interior, remains to be evaluated. Yet, increased denitrification throughout the ocean driven by particles can also explain the abundance of heterotrophic nitrogen fixers within particles and at depth given the resulting local nitrogen limitation necessitating diazotrophy to maintain growth^93,94^. Importantly, given that particles themselves are small and widely distributed, a dramatic nitrate deficit compared to phosphate indicative of denitrification, akin to the ODZs, is not to be expected. Interestingly given the experiments with intact and broken diatoms, the composition of a particle may not be strictly important for denitrification to occur as long as there remains some labile organic carbon. Nonetheless, to fully resolve the importance of marine particles for the fixed nitrogen budget, their complete size spectrum must be fully investigated for denitrification feasibility.

Nitrate and nitrite in these particles need not be fully reduced to dinitrogen, but rather the denitrification cascade could be halted at nitrous oxide (N_2_O) given the severe inhibition of oxygen for nitrous oxide reductase^77,95^. Moreover, in the real ocean, a particle is seeded by a diverse assemblage of organisms consisting not only of facultative denitrifiers but also autotrophic nitrifiers benefitting from the ammonification of the organic matter released from the phytoplankton. Aerobic nitrifiers too have been found to be associated with marine particles^96^, and anammox has been shown to rely on the ammonium released from organic matter degradation^97^. Particles would easily generate the low oxygen conditions that dramatically increase N_2_O yields during nitrification^98,99^, increasing denitrification rates by promoting nitrifier-denitrification.

While only one sinking speed and particle size was investigated here given the focus on microscale intraparticle development and the role of intact versus broken diatoms, these physical variables are of paramount importance in relating particle biogeochemistry to water column observations. As velocity and particle length scale control the boundary layer thickness, they have an important impact on the physiochemical gradients that separate the intraparticle microenvironment from the bulk water. Properly resolving biogeochemical impacts for the real ocean relies on integrating particle size spectra and each particle’s characteristic velocity across the three-dimensional ocean. Whereas such scaling is beyond the scope of this study, we have shown that the microscale dynamics associated with microcolony development opens up niches for anaerobic metabolisms within a few microns of a particle’s edge.

## Methods

### Isolation and phenotypic assay

Five nitrate-reducing heterotrophs—*Alterosomas* sp.; *Phaeobacter* sp.; *Idiomarina* sp.; *Virgibacillus* sp.; *Marinobacter* sp.—were isolated from subsurface seawater collected in the Sargasso Sea (collected and filtered in June 2014 at 40°67.4269′ N, 70°53.3153′ W, depth of 60.5 m, with a salinity of 33.16 and temperature of 11.7 °C), and from the phycosphere of our *Chaetoceros affinis* culture. First the seawater was enriched with filter sterilized (0.22 µm) diatom exudates for 5 days at room temperature (21 °C). 5 mL of diatom culture was centrifuged at 7000 rpm for 4 min at room temperature and resuspended in 500 µL of sterile seawater to concentrate the diatoms in a small volume. 100 µL of the enriched seawater and 100 µL of the diatom-concentrated seawater were then inoculated on agar plates (1% agar—VWR Agar for bacteriology) supplemented with Marine Broth 2216 (VWR) (MB) and incubated at room temperature (21 °C) for 5 days under aerobic conditions. Single colonies were collected and identified using 16S rRNA amplicon sequencing.

Each colony was resuspended in 5 mL of MB amended with 10 mM of nitrate, incubated for 48 h at room temperature under anaerobic conditions in a glove box (Coy Laboratory Products, Grass Lake, USA) with defined atmosphere, nitrogen (N_2_):hydrogen (H_2_) atmosphere (97:3). To determine their denitrification capability, first nitrite was measured with the standard colorimetric Griess reaction^100^. Second, we recorded nitrous oxide emissions (Supplementary Fig. 1) to confirm the complete denitrification capability of the community. We seeded agarose particles with the marine isolates (final OD = 0.0002) and incubated them under continuous flow of aerobically sparged (compressed air 10 mL min^−1^, volumetric flow of 0.01 mL min^−1^) MB in an air-tight millifluidic device. A nitrous oxide microsensor (Unisense, Aarhus, Denmark) with steel needle was inserted in the millifluidic outlet to record the dynamic production and consumption of nitrous oxide and confirm a complete denitrification pathway (Supplementary Fig. 1).

### 16S rRNA identification

Nineteen colonies isolated were resuspended in sterile PBS solution and the DNA was extracted using the DNeasy Powerlyzer Microbial Kit protocol (Qiagen, Hilden, Germany). After DNA extraction, the purity of the DNA was quantified with NanoDrop UV/Vis spectrophotometer (NanoDrop Technologies, Wilmington, DE). The DNA was then amplified with Quick-Load Taq 2X Master Mix (New England BioLabs, Ipswich, MA) and universal bacterial 8F/1492R primers. The samples were sent to Eton Bioscience for Sanger DNA sequencing using primers 8F and 907R. After the 16S rRNA identification and the phenotypic essay, 5 unique isolates at the genus level from the 19 candidates in total were obtained to use in the further experiments.

### Inocula preparation

*Chaetoceros affinis* and *Phaeodactylum tricornutum* were purchased from NCMA (Bigelow, National Center of Marine Algae and Microbiota, East Booth Bay, Maine). Once the diatom cultures arrived, they were immediately transferred to L1 media^101–104^. Prior to seeding the diatoms in the agarose, the diatom culture was grown for 5 days, *C. affinis* at 26 °C and *P. tricornutum* at 19 °C. The *C. affinis* intact diatoms were prepared in the following way. When the diatoms reached an OD of 1.5 at 320 nm, 5 mL of the culture was then filtered using a Falcon® 40 µm Cell Strainer to collect intact cells and resuspended in 1 mL of sterile seawater. 250 µL of *C. affinis* intact diatom suspension were finally inoculated in 1 mL of low melting agarose and gently vortexed to uniformly distribute them in the hydrogel. To obtain the broken diatom, 1 mL of diatom culture was sonicated for 1 min, power set at 60% (Branson Ultrasonics^TM^ Microtips for 200 Watt Sonifier; Branson Ultrasonics™ Sonifier™ SFX250/SFX550 Cell Disruptors). 250 µL of *C. affinis* broken diatom suspension were finally inoculated in 1 mL of low melting agarose and gently vortexed to uniformly distribute them in the hydrogel. The *P. tricornutum* diatoms were filtered using a Falcon® 40 µm Cell Strainer to collect intact cells and resuspended in 1 mL of sterile seawater. 250 µL of *P. tricornutum* suspension were finally inoculated in 1 mL of low melting agarose and gently vortexed to uniformly distribute the cells in the hydrogel.

Five bacterial species—*Alterosomas* sp.; *Marinobacter* sp.; *Phaeobacter* sp.; *Idiomarina* sp.; *Virgibacillus* sp.—were incubated overnight at 30 °C in 5 mL of Marine Broth 2216 (VWR) in a shaking incubator at 220 rpm. 200 µL of each overnight culture was resuspended in 20 mL of Marine Broth 2216 (VWR) in an Erlenmeyer flask in shaking incubator at 220 rpm for 3 h until each species reached an optical density of 0.15. 12 µL of each culture was inoculated into 1000 µL of low gelling agarose containing 10 µL of PyroScience oxygen nanoprobes (Oxnano; 10 mg mL^−1^) and diatoms.

*Pseudomonas aeruginosa* PAO1 NarK fusion reporter and *P. aeruginosa* PAO1 NirS fusion reporter^13^ were incubated in a shaking incubator at 220 rpm at 37 °C overnight. 200 µL of the overnight culture, was then resuspended in 20 mL of sterile LB media to reach 0.15 OD after 2 h of incubation in a shaking incubator at 220 rpm and 37 °C. 12 µL of each reporter strain was resuspended in low melting agarose containing 10 µL of Oxnano (10 mg mL^−1^) and diatoms with the same density as the marine isolates experiments described above.

### Millifluidic experiments

Once the low melting agarose was seeded with oxygen sensitive nanoparticles, bacteria, and diatoms, it was cast into 1.5 mm radius disks 700 µm in height and prepared as described previously^13^. The hydrogel particles were then enclosed in a millifluidic device as described before^13^, each containing four live seeded particles and one inert one upstream for calibration purposes. All the sinking particle experiments were performed with the same surface seawater (nominally 3 m depth), which was collected in October 2019 at 39°46.406′ N, 70°53.065′ W, bottom depth of 1578 m, with a salinity of 35.67, sea surface temperature of 23.3 °C, and below detection limit chlorophyll. The seawater was filter sterilized with a 0.20 µm vacuum filtration cup (VWR, 10040-468). In order to confirm the absence of any nitrate or nitrite, prior to performing the experiments, it was first tested for the presence of NO_x_^–^ species with the chemiluminescence method described below. The seawater was then amended with concentrated stocks of sodium phosphate monobasic (S3522-500G, Sigma Aldrich) to obtain a final concentration of 1 µmol L^−1^ and sodium nitrate (SS0680-500GR VWR) to obtain a final concentration of 10 µmol L^−1^.

The seawater was then transferred to acid-washed and autoclaved 125 mL serum bottles and sparged continuously with air (10 mL min^−1^). The volumetric flow through the millifluidic devices was maintained at a rate of 0.01 mL min^−1^ using a syringe pump (Harvard Apparatus PHD Ultra Syringe Pump), with 15 mL syringes (BD Luer-Lok tip) with in-line filter to sterilize the sample (Acrodisc® Syringe Filters with Supor® Membrane, Sterile - 0.2 µm, 25 mm). The seawater was sampled and stored in 40 mL VOA (ThermoFisher Scientific Catalog # 40-EPAVCSA). Each vial was washed with HPLC grade acetone (34850−1 L, Sigma Aldrich) and then combusted in an oven at 500 °C for 8 h. Milllifluidic devices and inlet and outlet tubes (Viton®, fluoroelastomer, High-Temperature Soft Rubber Tubing, Mc Master-Carr) were tested for the release of background dissolved carbon, and no detectable dissolved carbon was confirmed. Millifluidic devices with empty agarose particles were also tested for the release of background dissolved carbon, and no detectable dissolved carbon was found. Experiments were conducted in the dark, simulating the export of cells below the euphotic zone when they can no longer photosynthesize.

### Elemental carbon analysis

To monitor the change in total dissolved organic carbon (DOC), we used an Elementar Vario-EL analyzer modified to introduce liquid samples. We constructed our DOC calibration curve using a potassium hydrogen phthalate standard solution (LabCem, USA). Samples were analyzed within 5 h of collection. The samples were first filtered through 0.22 µm glass fiber filters (Kinesis KX, Canada) and diluted five times with in-lab produced reverse osmosis (Millipore Sigma, USA) water. The samples were then acidified with three drops of 37% hydrochloric acid (Acros Organics, USA) and analyzed. The analysis program included triplicate flush and injections with 0.2 mL per injection. After each run, a flush sequence was conducted to eliminate cross-contamination between samples.

To assess the carbon content in the initial diatoms used, we measured dry mass organic carbon using an Elementar Vario-EL analyzer modified to introduce solid sample equipped with a non-dispersive infrared detector (NDIR). Briefly, a small diatom sample was dispersed in 250 µL of seawater and pipetted into clean pre-weighed tin capsules (Elementar, Germany) to prepare for total solid organic carbon analysis. The capsules were allowed to dry in a 40 °C oven for 24 h. The samples were weighed again and the difference was computed. A blank was prepared to account for the seawater matrix. Last, the capsules were acidified with two drops of 12% hydrochloric acid (Acros Organics, USA) to liberate any inorganic carbon before analysis. Calibration curves were constructed using varying amounts of potassium hydrogen phthalate salt (Elementar, Germany). The total collected dry mass for broken and intact diatoms were 0.385 and 0.328 mg, respectively. On a per-particle basis, the broken diatom particles were seeded with 0.0585 mg of total carbon whereas the intact ones were seeded with 0.0497 mg of C per particle.

### Nitrate and nitrite measurements

The nitrate was measured using a standard chemiluminescence approach by quantifying the total NO_x_^–^ species via chemical reduction to NO, performed with hot acidified vanadium (III). Once the NO_x_^–^ is completely transformed to NO and quantified with a Teledyne T200 NOx analyzer^105,106^. Nitrite was quantified using 96 well plates with the Griess colorimetric assay^100^ and a plate reader (UV-Vis, Tecan, Spark) measuring the absorbance at 543 nm. 10 µL of combined Griess reagent (sulfanilamide and N-napthylethylenediamine) was added to 200 µL of seawater using the automatic injector module, incubated for 1 h at room temperature in the dark, and measured performing a lambda scan from 400 to 700 nm to obtain the full spectrum of absorbance for a more precise readout at sub-nanomolar concentrations (Supplementary Fig. 2).

### Image acquisition and analysis

All the images were acquired with a Nikon Eclipse Ti-2 microscope with an Andor Zyla 4.2 sCMOS VSC-06626 camera, 10× objective (Plan fluor), bright field and CY5 filter cube set (for the oxgen nanoprobe fluorescence), FITC for GFP and CY3 for dsRED. A time series of images at an interval of 1 h was continuously acquired until colonies stopped developing, for a total of at least 13 days. At each time point, a bright field image with a pseudo dark field setting was acquired together with the fluorescence image (excitation: 590–650 nm, emission: 662–737 nm) for the oxygen sensitive nanoparticles. CY3: excitation: 532–557 nm, emission 570–640 nm while FITC excitation 465–495 nm and emission from 525–515 nm. These two were only used on a separated experiment for the detection of the fluorescence reporters of Nar and Nir genes, respectively GFP and dsRed fluorescence proteins. All images were analyzed using MATLAB (R2016b) using the image analysis toolbox. At the end of each experiment, the particles were exposed to chloramphenicol (35–50 µg mL^−1^) to interrupt the metabolic activities of the bacteria. Seawater amended with chloramphenicol and sparged with N_2_ was flushed within the millifluidic device to obtain the anaerobic calibration of each particle. Multiple images were acquired, and using the NIS-Elements software, a profile of nanoparticle fluorescence across the particles was plotted during the image acquisition. When the fluorescence profile showed complete saturation of the nanosensor fluorescence (indicating full anoxia of the entire particle), these images were selected for the anaerobic calibration endmember. The same process was repeated, but with air-sparged media, to obtain the air-equilibrated particle calibration endmember. Notably, each particle has its own set of aerobic and anerobic images for calibration of the nanosensor to account for any heterogeneity in the nanoparticle distribution. Later, during the image analysis script, the calibration particles were divided into spatial zones as a function of radius to obtain a median value of the fluorescence nanosensor signal and create an optimal highly resolved local calibration (Fig. 1i, and Supplementary Fig. 3).

Using the elongation shape factor, corresponding to the square root of the ratio of the 2s moments of an object around its principal axes^107^, the diatoms were segmented and excluded from the fluorescence image in order to exclude their potential interference (by chlorophyll) with the oxygen nanoparticle signal (Supplementary Fig. 4a–d). A custom backtracking algorithm enabled us to follow each individual colony expansion while simultaneously quantifying the oxygen concentration change across the particle and within each individual microcolony (Supplementary Fig. 4e). The edge of the hydrogel particles was detected using the first image (Supplementary Fig. 4e). The images were enhanced using *imadjust* function, then using a Gaussian filter with a smoothing kernel with standard deviation of sigma = 20 followed by a global threshold and connected component analysis to fill the entire particle area (Supplementary Fig. 4e). Finally using the built-in function *regionprops*, the edge of the particle was identified. Using the built-in *edge* function followed by Otzu binarization. The binarized image is first dilated and then filled using a flood-fill operation, allowing bacterial colonies to be identified. For each individual colony, its position and distance from the particle edge was recorded (Supplementary Fig. 4e). For every colony at each time point, its cross-sectional area and median oxygen value (obtained using the local calibration values) was stored. The distribution of the signal of the oxygen nanosensor of each colony and of the particle excluding the colonies was stored as equivalent oxygen values (obtained using the local calibration values) at each time point (Fig. 1, Supplementary Fig. 3).

### Field work

A cruise was conducted in the Eastern Tropical North Pacific oxygen deficient zone aboard the *R/V Falkor* (cruise FK180624) in June and July 2017. The cruise transited along the 14°N latitude parallel. Oxygen, fluorescence, temperature, salinity, and beam attenuation were determined directly from the CTD package with in-house calibrations. Density was calculated from temperature, salinity, and pressure using the TEOS−10 equation of state. Nitrite concentrations were measured with the standard Griess assay, details of which can be found elsewhere^108^.

### Reporting summary

Further information on research design is available in the Nature Portfolio Reporting Summary linked to this article.

### Supplementary information

Supplementary Information

Reporting Summary

## Data Availability

The image analysis and analytical chemistry data are available at BCO-DMO (Project number 897881) and at Zenodo, 10.5281/zenodo.8126751. Cruise data can be found at BCO-DMO (10.26008/1912/bco-dmo.832389.1).
