# Supplementary material for: Microscale dynamics promote segregated denitrification in diatom aggregates sinking slowly in bulk oxygenated seawater
Source: Commun Earth Environ. 2023 Jul 28;4(1):275. doi: 10.1038/s43247-023-00935-x (PMC11041763; doi:10.1038/s43247-023-00935-x)
Supplement: Supplementary file 1 — Supplementary Information [file 43247_2023_935_MOESM1_ESM.pdf]

Supplementary materials for

Microscale dynamics promote segregated denitrification in diatom aggregates sinking slowly in bulk oxygenated seawater

AUTHORS: Davide Ciccarese[1*,] Omar Tantawi[2], Irene H. Zhang[1,3], Desiree Plata[2], Andrew R. Babbin[1*]

AFFILIATIONS: [1]Department of Earth, Atmospheric & Planetary Sciences, Massachusetts Institute of Technology, Cambridge, MA, USA; [2]Department of Civil and Environmental Engineering, Massachusetts Institute of Technology, Cambridge, MA, USA; [3]Program in Microbiology, Massachusetts Institute of Technology, Cambridge, MA, USA

* Corresponding authors (davidec@mit.edu ; babbin@mit.edu)

Table of contents:

1. Supplementary Fig. 1 | Nitrous oxide quantification.
2. Supplementary Fig. 2 | Nitrite quantification with plate reader.
3. Supplementary Fig. 3 | Aerobic and anaerobic calibration of in situ oxygen nanoprobes in the particles.
4. Supplementary Fig. 4 | Step-by-step image analysis algorithm.
5. Supplementary Fig. 5 | Oxygen change at colony level of particles seeded with broken diatoms.
6. Supplementary Fig. 6 | Oxygen change at particle level of particles seeded with broken diatoms.
7. Supplementary Fig. 7 | Oxygen change at colony level of particles seeded with intact diatoms.
8. Supplementary Fig. 8 | Oxygen change at particle level of particles seeded with intact diatoms.
9. Supplementary Fig. 9 | Spatial expression of *narK* and *nirS* genes for experiment with *Phaeodactylum tricornutum*.
10. Supplementary Fig. 10 | Nitrite evolution in bulk water around particles seeded with *Pseudomonas aeruginosa* and *Phaeodactylum tricornutum* or *Chaetoceros affinis*.
11. Supplementary Fig. 11 | Nitrate evolution in surrounding water.
12. Supplementary Fig. 12 | Nitrite evolution in surrounding water.
13. Supplementary Fig. 13 | Dissolved organic carbon (DOC) evolution in surrounding water.

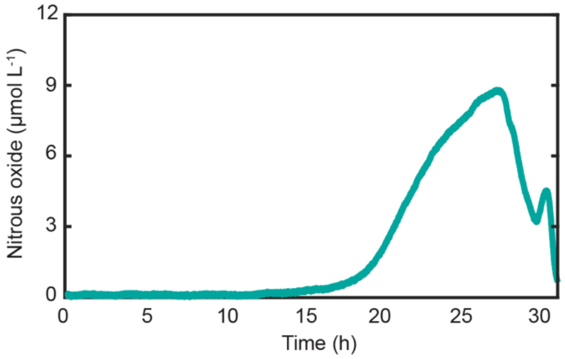

Supplementary Fig. 1 | Nitrous oxide quantification. Nitrous oxide production and consumption of four particles seeded with the marine isolates used in this study, confirming denitrification capability. The signal was measured with a microsensor inserted at the outlet of the millifluidic device. The media for this test was Marine Broth 2216 amended with 400 µmol L^{-1} of nitrate.

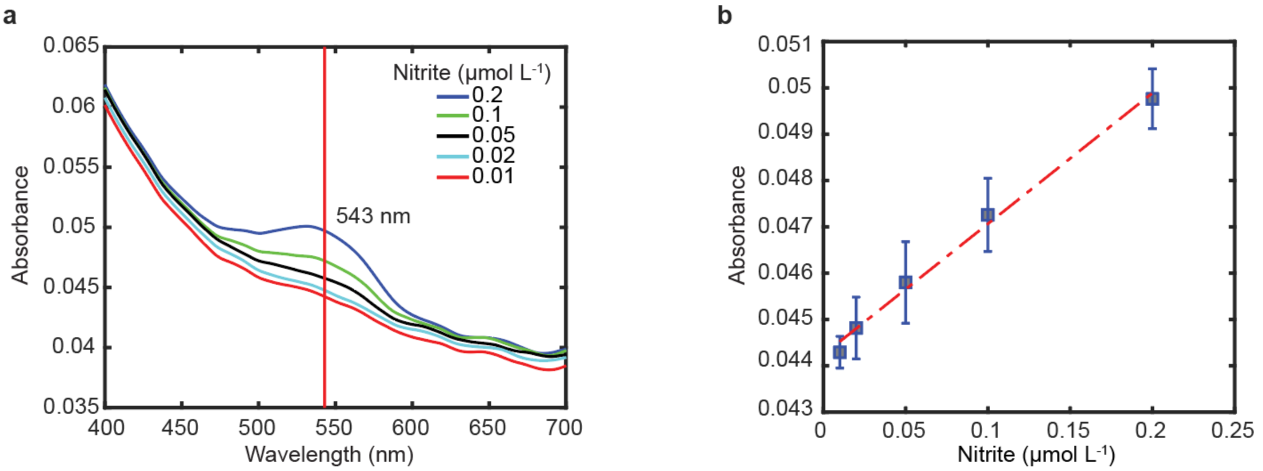

Supplementary Fig. 2 | Nitrite quantification with plate reader. a) Mean signal of five technical replicates of lambda scan 400–700 nm. The red line indicates the 543 nm peak, corresponding to the maximum absorbance of Griess test solution. **b)** The squares represent mean value of five technical replicates of absorbance peaks; the error bars represent the standard deviation. The linear best fit equation is shown with the dashed red line.

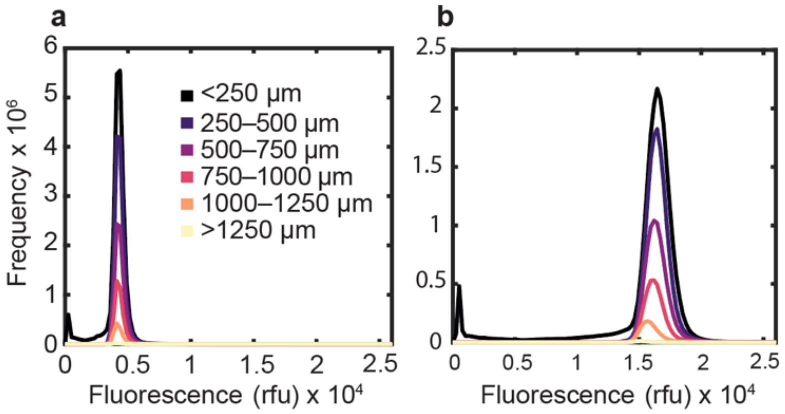

Supplementary Fig. 3 | Aerobic and anaerobic calibration of in situ oxygen nanoprobes in the particles. A representative particle is shown for **a)** aerobic and **b)** anaerobic calibration of oxygen fluorescence (in relative fluorescence units, rfu). Each line is color coded based on a defined zone as a function of spatial location in the particle on the basis of distance from the edge of the particle: black, <250 µm from the edge; dark purple, 250–500 µm; magenta, 500–750 µm; red, 750–1000 µm; orange, 1000–1250 µm; and yellow, >1250 µm from the particle's edge (i.e., closest to the center). Each zone's median value of the fluorescence nanosensor signal is used to create an optimal highly resolved local calibration.

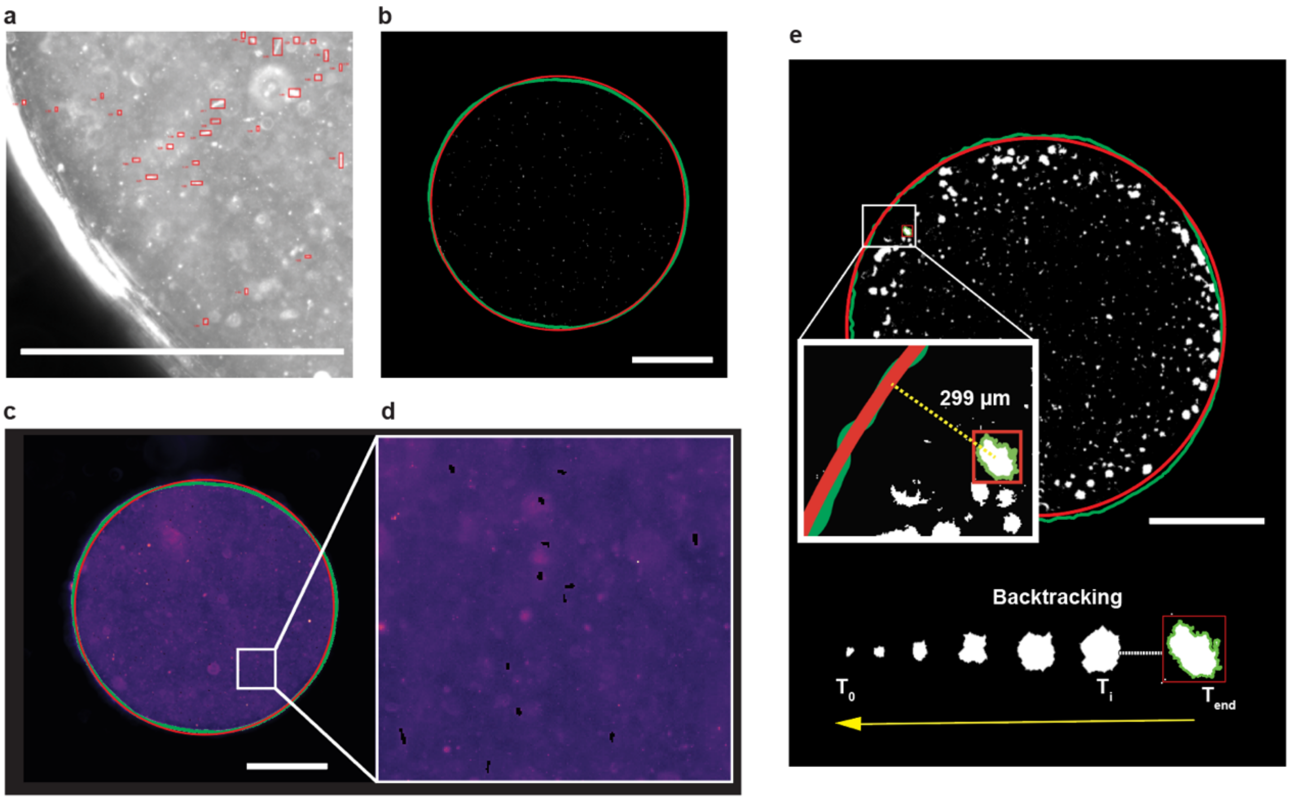

Supplementary Fig. 4 | Step-by-step image analysis algorithm. a) Diatoms are recognized using an elongation shape factor. **b)** Binarized image of segmented diatoms. The edge of the hydrated particle is segmented and the contours are highlighted in green. In red is the fitted circle on the edge of the hydrated particle. **c)** Oxygen-sensing nanoparticles image across a full particle. The diatoms are subtracted from the fluorescence image to avoid interference by chlorophyll. **d)** Close up of diatoms subtracted from the fluorescence image. **e)** Each individual segmented colony is identified within a bounding box, and the distance from the edge of the hydrogel particle and area of the colony is recorded at each time point with a backtracking algorithm from the final measurement (the example shown is 299 μm from the center of the colony to the nearest particle edge). The white scale bar in all panels denotes 1000 μm.

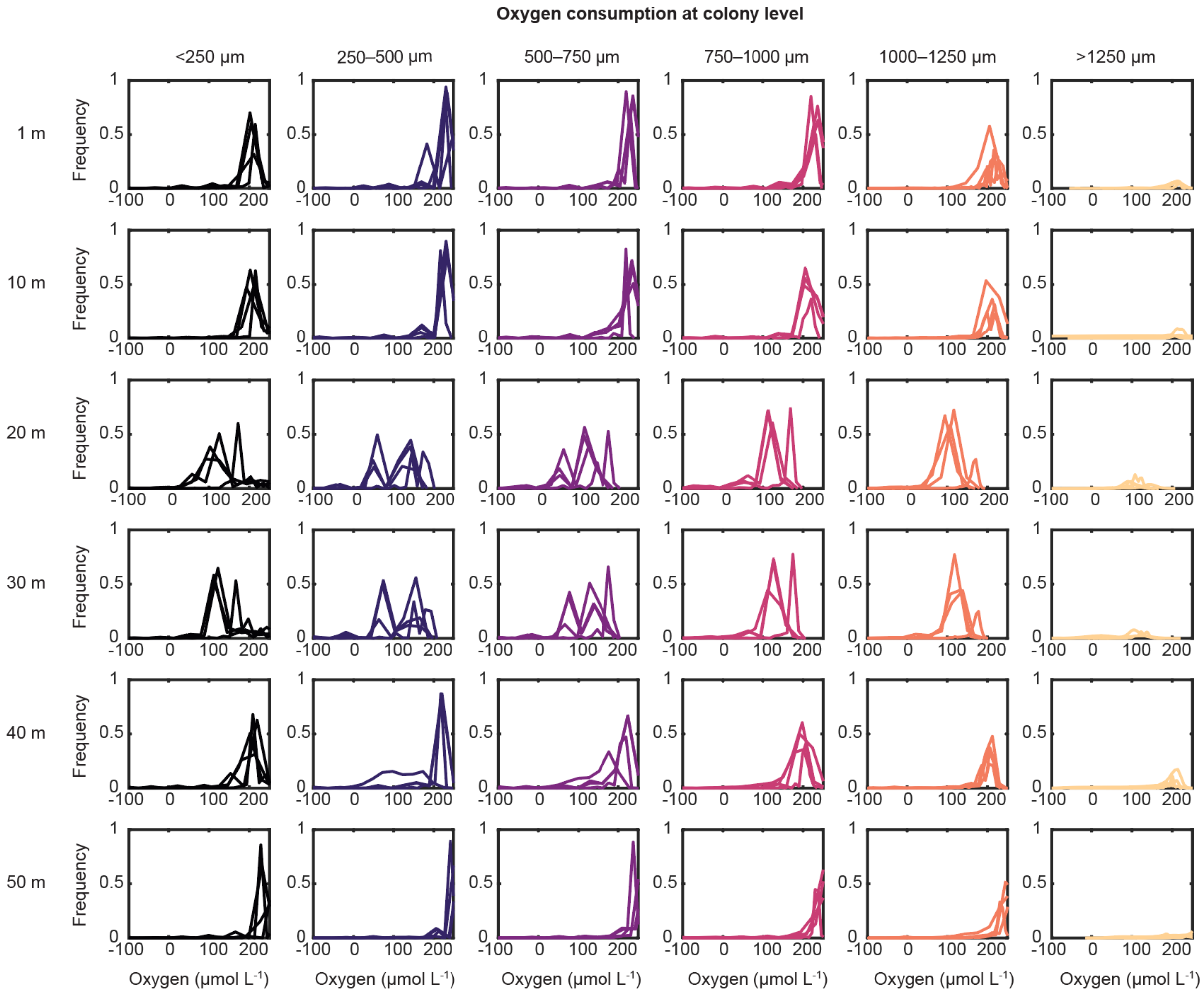

Supplementary Fig. 5 | Oxygen change at colony level of particles seeded with broken diatoms. At each time point translated to depth (rows), the kernel density estimate represents the probability density with normalized values set to 1, showing the fluorescence signal of oxygen in all six spatial zones (columns) of all the colonies within each zone. Replicate particles: n = 4.

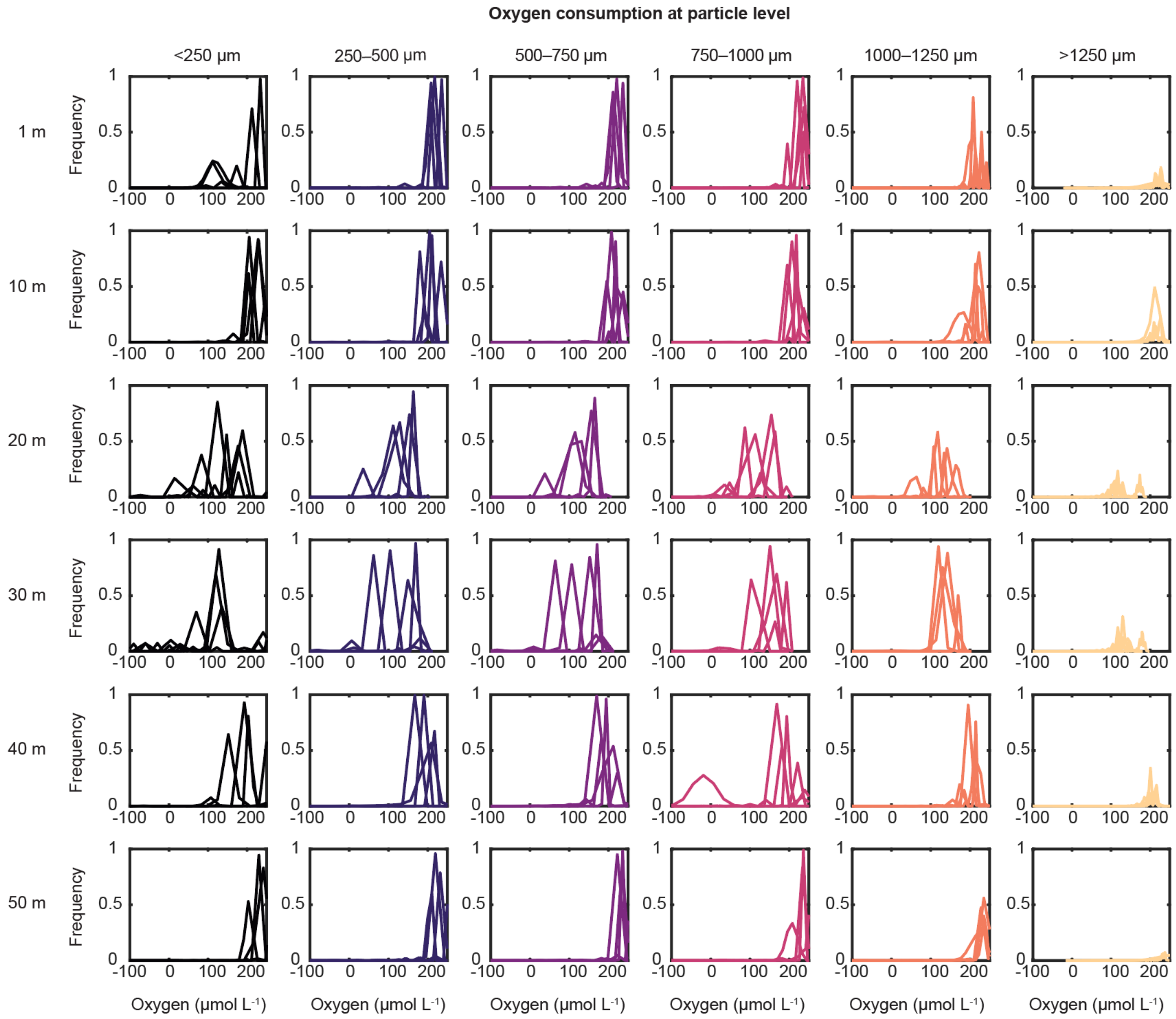

Supplementary Fig. 6 | Oxygen change at particle level of particles seeded with broken diatoms. At each time point translated to depth (rows), the kernel density estimate represents the probability density with normalized values set to 1, showing of the fluorescence signal of oxygen in all six spatial zones (columns) of the particle excluding the colonies. Replicate particles n = 4.

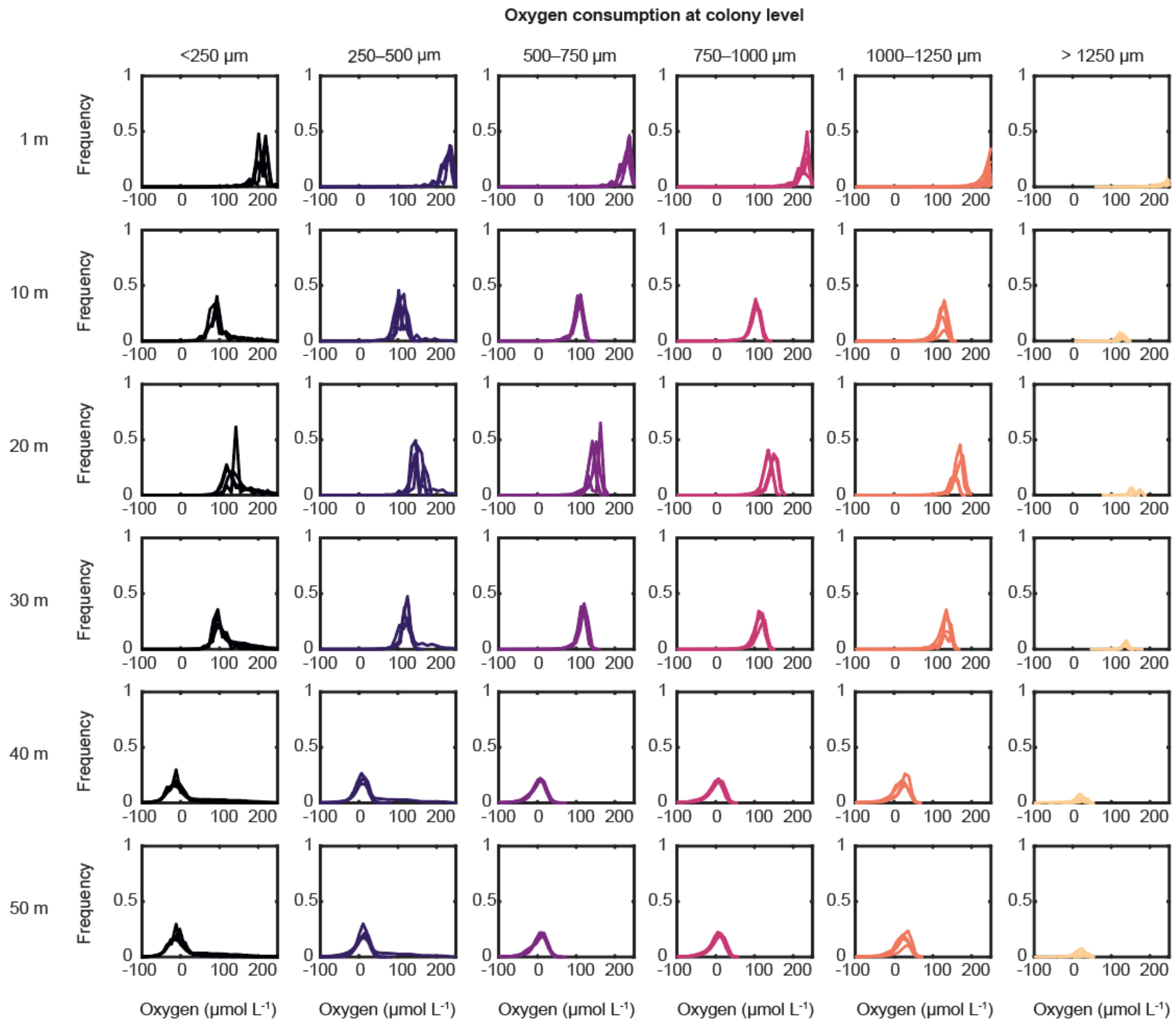

Supplementary Fig. 7 | Oxygen change at colony level of particles seeded with intact diatoms. At each time point translated to depth (rows), the kernel density estimate represents the probability density with normalized values set to 1, showing the fluorescence signal of oxygen in all six spatial zones (columns) of all the colonies within each zone. Replicate particles n = 4.

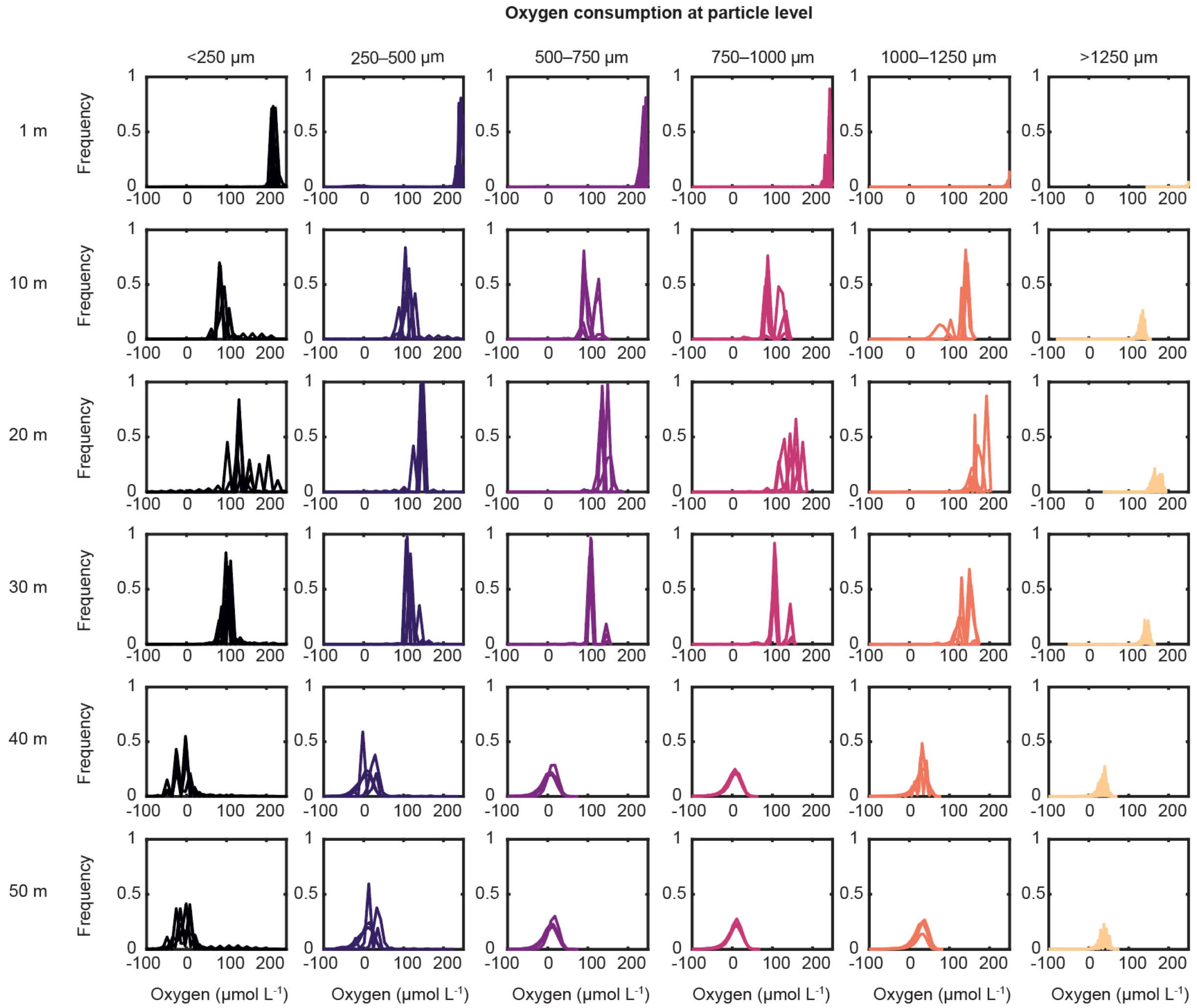

Supplementary Fig. 8 | Oxygen change at particle level of particles seeded with intact diatoms. At each time point translated to depth (rows), the kernel density estimate represents the probability density with normalized values set to 1, showing of the fluorescence signal of oxygen in all six spatial zones (columns) of the particle excluding the colonies. Replicate particles n = 4.

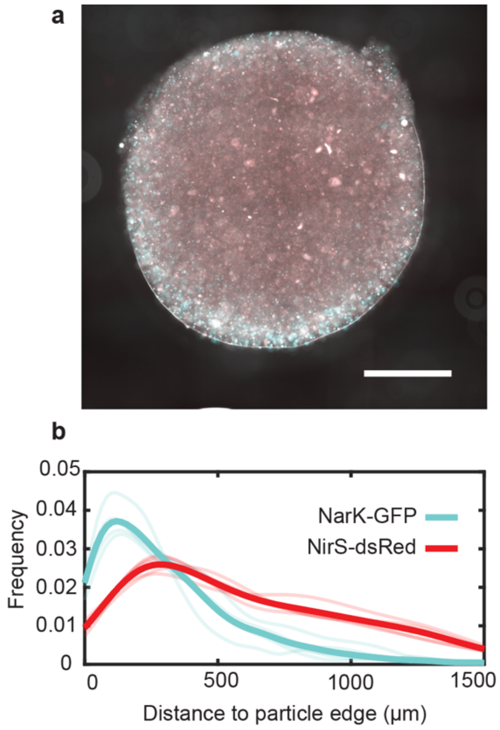

Supplementary Fig. 9 | Spatial expression of *narK* and *nirS* genes for experiment with *Phaeodactylum tricornutum*. a) Fluorescence microscopy image of a representative particle seeded with *Pseudomonas aeruginosa* PAO1 NarK-GFP, PAO1 NirS-dsRed, and *Phaeodactylum tricornutum*. NarK-GFP is shown in cyan whereas NirS-dsRed and native chlorophyll fluorescence are in red. White scale bar denotes 1000 μm. **b)** Probability density functions of the expression of NarK-GFP and NirS-dsRed reporters of five particles as a function of distance from the particle edge, with the average shown as the thick curve.

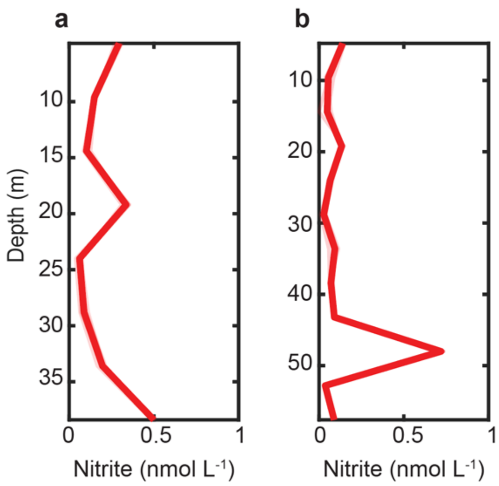

Supplementary Fig. 10 | Nitrite evolution in bulk water around particles seeded with *Pseudomonas aeruginosa* and *Phaeodactylum tricornutum* or *Chaetoceros affinis*. a) Nitrite measured in the bulk flowing seawater around 4 particles seeded with *P. aeruginosa* PAO1 NarK-GFP, *P. aeruginosa* PAO1 NirS-dsRed, and *P. tricornutum*. 5 technical replicates are shown with the thick red curve indicating the mean. **b)** Same as **a** but using *C. affinis* as the diatom carbon source.

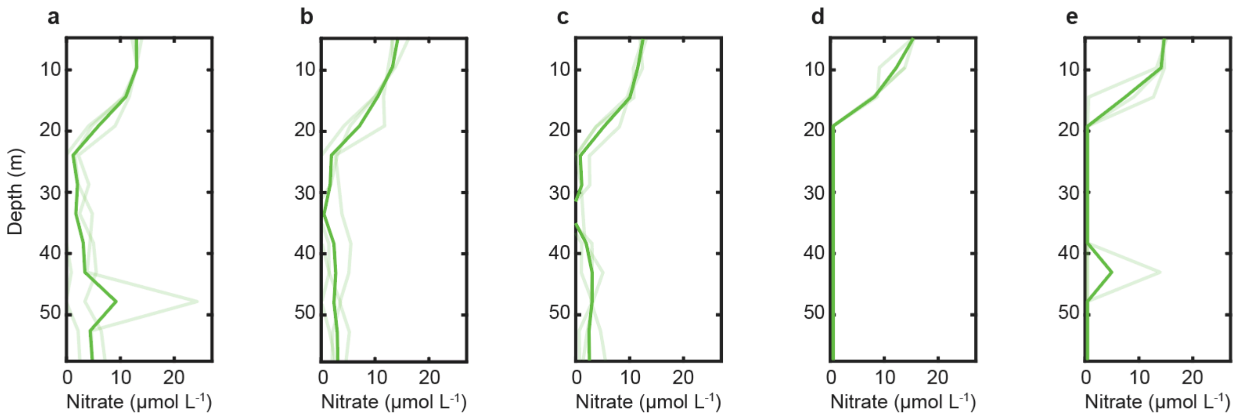

Supplementary Fig. 11 | Nitrate evolution in surrounding water. Nitrate profiles measured in the water flowing around particles seeded with **a)** *Pseudomonas aeruginosa* PAO1 NarK-GFP, PAO1 NirS-dsRed and broken *Chaetoceros affinis*, **b)** broken *Chaetoceros affinis* carrying xenic bacteria, **c)** only with marine bacteria, **d)** *Pseudomonas aeruginosa* PAO1 NarK-GFP, PAO1 NirS-dsRed and intact *Chaetoceros affinis*, or **e)** intact *Chaetoceros affinis* carrying xenic bacteria. Each panel shows 3 separate millifluidic device replicates each containing 4 replicate particles, with the mean displayed in darker green.

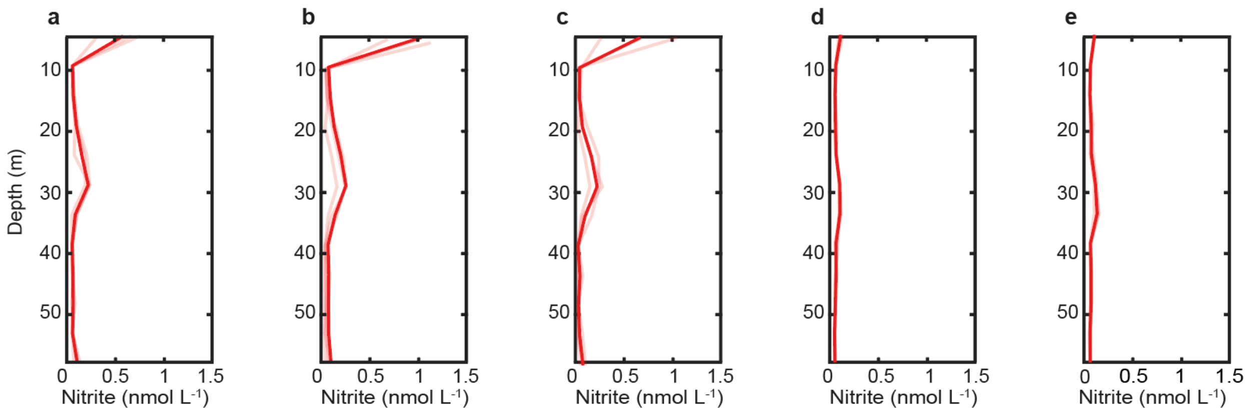

Supplementary Fig. 12 | Nitrite evolution in surrounding water. Nitrite profiles measured in the water flowing around particles seeded with **a)** *Pseudomonas aeruginosa* PAO1 NarK-GFP, PAO1 NirS-dsRed and broken *Chaetoceros affinis*, **b)** broken *Chaetoceros affinis* carrying xenic bacteria, **c)** only with marine bacteria, **d)** *Pseudomonas aeruginosa* PAO1 NarK-GFP, PAO1 NirS-dsRed and intact *Chaetoceros affinis*, or **e)** intact *Chaetoceros affinis* carrying xenic bacteria. Each panel shows 3 separate millifluidic device replicates each containing 4 replicate particles, with the mean displayed in darker red.

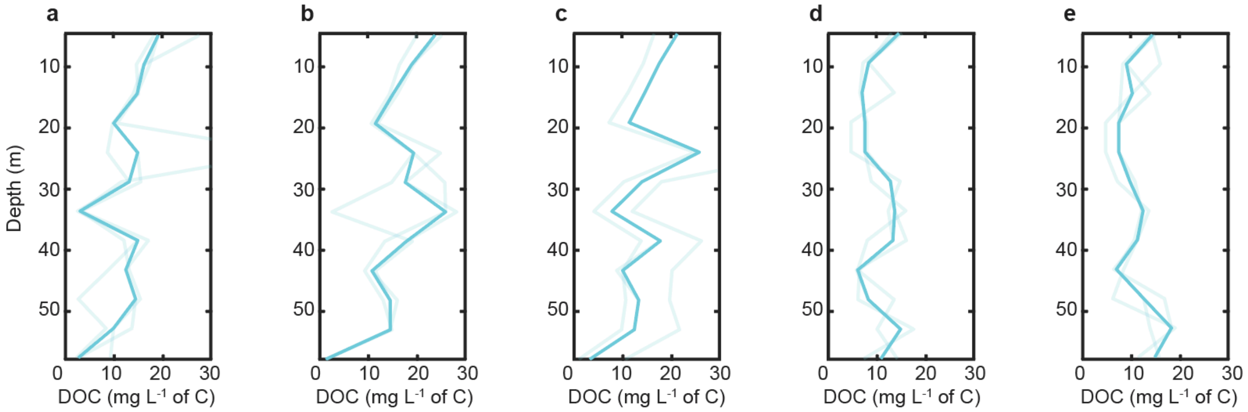

Supplementary Fig. 13 | Dissolved organic carbon (DOC) evolution in surrounding water. DOC profiles measured in the water flowing around particles seeded with **a)** *Pseudomonas aeruginosa* PAO1 NarK-GFP, PAO1 NirS-dsRed and broken *Chaetoceros affinis*, **b)** broken *Chaetoceros affinis* carrying xenic bacteria, **c)** only with marine bacteria, **d)** *Pseudomonas aeruginosa* PAO1 NarK-GFP, PAO1 NirS-dsRed and intact *Chaetoceros affinis*, or **e)** intact *Chaetoceros affinis* carrying xenic bacteria. Each panel shows 3 separate millifluidic device replicates each containing 4 replicate particles, with the mean displayed in darker blue.
